# Barrier Perturbation in Porcine Peyer’s Patches by Tumor Necrosis Factor is Associated With a Dysregulation of Claudins

**DOI:** 10.3389/fphys.2022.889552

**Published:** 2022-05-30

**Authors:** Linda Droessler, Valeria Cornelius, Elisa Boehm, Laura Stein, Nora Brunner, Salah Amasheh

**Affiliations:** Institute of Veterinary Physiology, Department of Veterinary Medicine, Freie Universität Berlin, Berlin, Germany

**Keywords:** epithelial barrier, claudins, inflammation, peyer’s patch, tight junction, TNF, TNFR, ussing chamber

## Abstract

The proinflammatory cytokine tumor necrosis factor (TNF) has been described as one of the main mediators of intestinal inflammatory diseases, affecting the composition of tight junction (TJ) proteins and leading to a disruption of the epithelial barrier. An intact intestinal barrier is mandatory, because the follicle-associated epithelium of Peyer’s patches represents the first defense line of the intestinal immune system and ensures a controlled uptake of antigens from the gut lumen. In the current study, we have analyzed the detailed effects of TNF on the follicle-associated epithelium of porcine Peyer’s patches by applying the Ussing chamber technique. Epithelial tissue specimens of Peyer’s patches and the surrounding villus epithelium were mounted into conventional Ussing chambers and incubated with TNF for 10 h. The transepithelial resistance, representing epithelial barrier function of the tissue, was recorded. A reduction of transepithelial resistance was detected after 8 h in Peyer’s patch tissue specimens, whereas the villus epithelium was not significantly affected by TNF. Subsequent molecular analysis of TJ protein expression revealed a marked decrease of claudin-1 and -4, and an increase of claudin-2. In neighboring villus epithelium, no significant changes in the expression of TJ proteins could be shown. A strong increase of TNF receptor-2 (TNFR-2) could also be detected in Peyer’s patches, in agreement with the major role of this receptor in Peyer’s patches. Our findings were in accordance with changes detected by confocal laser scanning immunofluorescence microscopy. The regulation of TNF effects via myosin light chain kinase (MLCK) was analyzed in blocking experiments. Our detailed analysis is the first to show that TNF affects the barrier function of the follicle-associated epithelium of porcine Peyer’s patches but has no effects on the villus epithelium. These findings reveal not only the basic differences of epithelial barrier function between the two structures, but also the significance of Peyer’s patches as a primary mucosal immune defense.

## Introduction

The intestine is exposed to a multitude of exogenous substances, and therefore an efficient epithelial barrier is mandatory for limiting paracellular permeability. As a major part of the gut-associated lymphatic tissue (GALT), Peyer’s patches (PP) are located in the distal small intestine and hence represent a first line of immunological defense (Neutra et al., 2001). It consists of three main components: the follicular and interfollicular area, containing the germinal center with macrophages, dendritic cells or proliferating B-lymphocytes, and the follicle-associated epithelium (FAE) (Jung et al., 2010). The FAE, which covers PP, is the main component building a functional barrier of the lymphatic tissue against the gut lumen. In comparison with the surrounding villus epithelium (VE), the FAE lacks mucin-secreting goblet cells but contains M cells, which are responsible for the uptake and presentation of specific antigens to the lymphoid follicle (Sakhon et al., 2015). Furthermore, the FAE also differs from the VE with regard to the expression of tight junction (TJ) proteins, which enable a more restrictive sealing of the paracellular pathway in accordance with the specific immunological role of Peyer’s patches (Markov et al., 2016; Radloff et al., 2017).

Tumor necrosis factor (TNF) is released by macrophages and can subsequently mediate the production of other cytokines, which is the reason that it is described as a proinflammatory cytokine (Ruder et al., 2019). It plays a crucial role in the pathogenesis of infections, inflammation, and apoptosis, and is one of the main components in the pathogenesis of inflammatory bowel diseases (IBDs) (Barbara et al., 1996; Gibson, 2004; Schulzke et al., 2009). By disturbing TJ proteins, TNF is able to affect the epithelial barrier function, resulting in a decreased transepithelial resistance and an increased permeability to solutes as initially shown in cultured renal epithelial cells (Mullin and Snock, 1990). The decreased expression of tightening TJ proteins and an increased expression of the pore-forming TJ protein claudin-2 can be observed in intestinal inflammatory diseases, such as Crohn’s disease or ulcerative colitis (Zeissig et al., 2007; Oshima et al., 2008; Amasheh S. et al., 2009). In light of general immunological questions, the study of porcine models has recently gained major attention. Moreover, a closer look at porcine gut pathophysiology is of major interest, as intestinal diseases have been shown to lead to drastic economic consequences. For example, post weaning diarrhea (PWD) is a common disease in the swine industry, often leading to sudden death within 2 weeks after weaning (Fairbrother et al., 2005). Recent studies have revealed a major function of TNF in the pathogenesis of PWD, as increased expression levels have been observed both *in vivo* and *in vitro* (Tang et al., 2020; Wu et al., 2021). However, the effects of TNF on the FAE of porcine PP have not been analyzed as yet.

In our current study, we hypothesized the functional and molecular difference of PP compared to VE under inflammatory conditions. Based on our previous findings (Droessler et al., 2021), we have aimed at analyzing the effect of TNF on paracellular barrier function in the porcine PP over 10 h. Therefore, an optimized Ussing chamber protocol has been applied to ensure the vitality of the tissue over time. The Ussing chamber technique is widely used for measurements of ion or drug transport in intestinal epithelia and provides an essential method for electrophysiological analysis (Amasheh et al., 2004; Westerhout et al., 2015). Tissue samples have been further processed for the analysis of TJ composition and regulation.

## Materials and Methods

### Tissue Preparation

Tissue specimens from the small intestine with and without PP from 28 adult pigs were taken immediately after slaughter at a slaughterhouse (Lehr-und Versuchsanstalt für Tierzucht und Tierhaltung, Ruhlsdorf, Germany). The tunica serosa and muscularis externa were stripped off the mucosa, and the remaining tissue was rinsed with 0.9% NaCl to remove chyme and mucus. The tissue samples were transported to the laboratory in ice-cold buffer containing (in mmol/L): Na^+^ (149.4), Cl^−^ (128.8), K^+^ (5), Ca^2+^ (1.2), Mg^2+^ (1.2), HCO_3_^−^ (25), H_2_PO_4_^−^ (0.6), HPO_4_^2-^ (2.4), d-Glucose (10) (all from Carl Roth GmbH, Karlsruhe, Germany) and Enrofloxacin (30 μmol/L; MP Biomedicals, Eschwege, Germany). The buffer was gassed with 95% O_2_ and 5% CO_2_, and the pH was adjusted to 7.4 before transport.

### Ussing Chamber Experiments and TER Measurements

**FIGURE 1:**
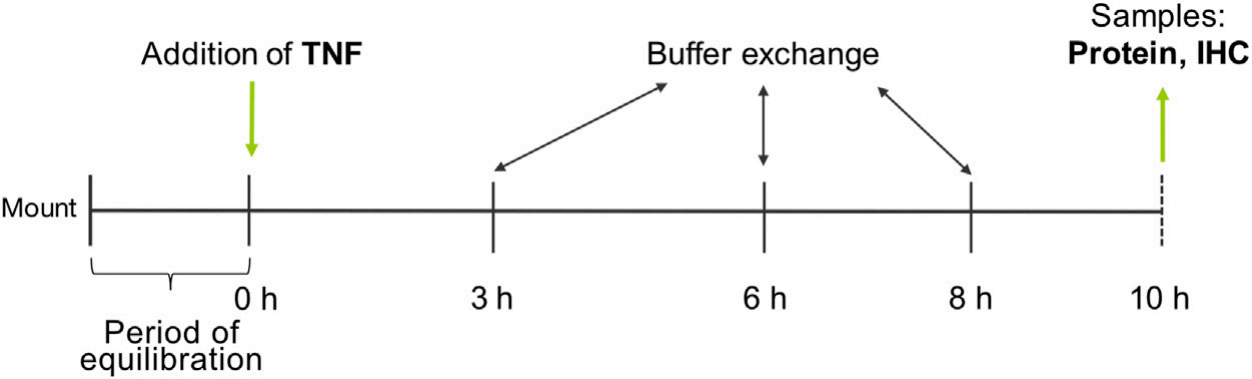
Experimental setup.

PP and VE of the tissue specimens were differentiated visually and mounted into conventional Ussing chambers equipped with gas lifts. The bathing solution was equivalent to the transport buffer and was constantly gassed with 95% O_2_ and 5% CO_2_ at a pH of 7.4. The area of the tissue exposed to the buffer was 0.96 cm^2^. After an equilibration period of 60 min, TNF was added at three different concentrations (1000, 5000, or 10,000 U/ml) to the serosal side of the tissue, and TER was recorded permanently for 10 h. A buffer exchange was carried out after 3, 6, and 8 h to ensure the viability of the tissue (Figure 1).

### Permeability Measurements

Unidirectional paracellular tracer flux measurements from the mucosal to the serosal side were carried out under voltage-clamp conditions for the higher TNF concentrations (5000, 10,000 U/ml). After 8 h of incubation with the cytokine, 2 μCi [^3^H]-D-Mannitol (182 Da; PerkinElmer, Waltham, MA, United States) was added to the mucosal side, and samples were taken directly after addition of the tracer and at the end of the flux periods from the same side. Samples of 600 μl were taken from the basolateral side every 40 min, giving three flux periods over 2 h. After removal of the samples, the absent volume was refilled with 600 μl fresh buffer containing the corresponding TNF concentration. Subsequently, Aquasafe 300 plus liquid scintillation cocktail (Zinsser Analytics, Frankfurt, Germany) was added, and each sample was analyzed using a TriCarb 4910 TR liquid scintillation counter (PerkinElmer, Waltham, MA, United States). Calculation of the specific activity and paracellular flux of the tracer was carried out as described previously (Droessler et al., 2021).

### Fixation and Processing of Tissue Samples

Following the Ussing chamber experiments, tissue samples were fixed at room temperature in 4% paraformaldehyde (PFA) in PBS containing Mg^2+^ and Ca^2+^ (PBS +/+) for 4 h. Subsequently, the samples were washed once in PBS +/+ and transferred into 25 mM glycine in PBS +/+ containing 0.1% NaN_3_. Tissue samples were then stored at 4°C until the staining step. For dehydration, the samples were first transferred into increasing alcohol concentrations (from 70 to 100%), then into xylol, and finally into paraffin for embedding.

### Hematoxylin-Eosin Staining

For analysis of tissue quality, samples of VE and PP were taken after 10 h of incubation with or without TNF. Cross-sectioning, deparaffinization, and rehydration were carried out prior to staining. Samples were stained for 3 min with hematoxylin solution according to Harris (Carl Roth GmbH, Karlsruhe, Germany), rinsed in 0.1% hydrochloric acid, and differentiated under flowing water for 3–5 min. The sections were then stained with eosin Y solution (1%; Carl Roth GmbH, Karlsruhe, Germany) for another 3 min, washed with water, and again transferred into increasing alcohol concentrations with a final step of xylol as explained above. Slides were subsequently embedded with ProTaqs paramount (Biocyc, Luckenwalde, Germany).

### Protein Extraction and Quantification

Subsequent to the Ussing chamber experiments, tissue samples were frozen in liquid nitrogen and stored at −80°C. For protein extraction, the specimens were homogenized in RIPA buffer containing 25 μM HEPES pH 7.6, 25 μM NaF, 2 μM EDTA, 1% SDS (10%), H_2_O, and enzymatic protease inhibitors (Complete EDTA-free, Boehringer, Mannheim, Germany). The samples were then centrifuged for 1 min at 16,000 × g, and the supernatant was left for 30 min on ice for further lysis. A second centrifugation step for 15 min at 15,000 × g at 4°C (sigma 3–30 ks, Sigma-Aldrich, Munich, Germany) was carried out, and the supernatant was transferred into Eppendorf tubes. The Bio-Rad DC Protein Assay (Bio-Rad Laboratories GmbH, Munich, Germany) was used to quantify the proteins, which were detected by an EnSpire Multimode Plate Reader (Perkin Elmer, Waltham, MA, United States).

### Immunoblotting and Densitometry

TJ proteins in FAE of porcine PPs after incubation with TNF in Ussing chambers were analyzed by primary antibodies raised against claudin-1, -2, -3, -4, -7, and occludin (Life Technologies, Carlsbad, California, United States). For the detection of claudin-2, urea (9 mol/L, Carl Roth GmbH, Karlsruhe, Germany) was added, and samples were denatured at 55°C for 8 min to ensure the unlocking of the hydrogen bonds. Expression of the specific receptors in VE and PP subsequent to incubation with TNF was examined using primary antibodies raised against TNFR-1 (abcam, Berlin, Germany) and TNFR-2 (antibodies-online GmbH, Aachen, Germany). Immunoblotting was performed as described previously (Droessler et al., 2021). Densitometry was carried out by normalizing protein bands to the amount of total protein and TNF-treated groups were compared with controls, respectively.

### Immunohistochemistry

For immunohistological staining, tissue samples were first rehydrated in reverse order as described in Fixation and Processing of Tissue Samples. Subsequently, epitopes were exposed by boiling the samples in EDTA buffer (pH 8) for 45 min, followed by a permeabilization step in Triton X-100 in PBS +/+ for 5 min at room temperature. The tissue sections were framed using a PAP pen (Kisker Biotech GmbH & Co. KG, Steinfurt, Germany) to provide a hydrophobic barrier and were blocked afterwards for 30 min at room temperature in PBS containing 5% goat serum and 1% bovine serum albumin. Samples were then incubated with primary antibodies raised against claudin-1, -2, -4, occludin, ZO-1 (Thermo Fisher Scientific), TNFR-1 (abcam, Berlin, Germany), and TNFR-2 (antibodies-online GmbH, Aachen, Germany) for 1 h at 37°C, followed by four washing steps in blocking solution. Subsequently, secondary goat anti-rabbit Alexa Fluor-488, goat anti-mouse Alexa Fluor-594, and DAPI for the staining of nuclei were added to the samples, which were incubated again for 1 h at 37°C. Another four washing steps in blocking solution were carried out, followed by one wash in distilled water, and finally the sections were mounted in ProTaqs Mount Fluor (Biocyc, Luckenwalde, Germany). Microscopic analysis was carried out using a Zeiss 710 confocal microscope (Zeiss, Oberkochen, Germany).

### Signaling Experiments With ML-7

Experimental approaches with ML-7, a specific blocker of the myosin light chain kinase (MLCK), were subsequently carried out to determine whether the observed changes in TER were related to MLCK signaling. PP tissue samples from eight animals were processed as described in Tissue Preparation. TNF was added at a concentration of 5000 U/ml to the serosal side of the tissue. ML-7 (Sigma Aldrich, Munich, Germany) was dissolved in dimethyl sulfoxide (DMSO; Sigma Aldrich, Munich, Germany), corresponding to a final concentration of 7.5 μM, and was added simultaneously with TNF to the mucosal and serosal sides. Controls were treated with the respective amount of DMSO. Again, TER was recorded for 10 h, and buffer exchanges were carried out as described previously.

### Testing of Vitality

After 10 h in the Ussing chamber, the vitality of the tissues was examined. Therefore, 4 ml buffer was removed from both sides of the Ussing chamber and replaced with 4 ml of fresh buffer containing theophylline (8 mM; Sigma Aldrich, Munich, Germany). The theophylline challenge was carried out for 10 animals, respectively.

### Cell Culture Experiments

We used IPEC-J2 cells (DSMZ, Braunschweig, Germany), a non-transformed cell line that stems from porcine jejunal epithelia, to analyze whether the changes in epithelial barrier function in PP tissue also occur in a cell culture model. Cells were seeded on semi-permeable cell culture inserts (Millipore, Darmstadt, Germany) at 10^5^ cells per filter. Dulbecco’s MEM/Ham’s F12 with 3.15 g/L glucose and 2 mM stable glutamine (Biochrom, Berlin, Germany) was used and supplemented with 10% porcine serum and 1% penicillin/streptomycin (Sigma Aldrich, Munich, Germany). Medium was changed every 2–3 days, and filters were filled with 500 μl apically and 1 ml basolaterally. By using a chopstick electrode and an epithelial Volt/Ohm Meter (EVOM, World Precision Instruments, Sarasota, FL, United States), we measured the TER immediately before each media exchange, and values were corrected with the media and blank values used in our experimental settings. Once the cells had built up a confluent monolayer, resulting in consistent TER values, the incubation experiments were started. We added 5000 U/ml TNF (PeproTech, Hamburg, Germany) to the basolateral side of the cell filters, and TER was recorded for up to 10 h. Cell passages between 8 and 13 were used for experiments.

### Statistical Analysis

The data are expressed as means and standard error of the mean (SEM). For *in vitro* experiments, n is the number of cell filters, whereas for *ex vivo* experiments, n represents the number of animals used. For TER measurements, statistical analysis was performed using one-way ANOVA and Dunnett’s test for the correction of multiple testing for normally distributed data and Kruskal Wallis test for non-normally distributed data. Student’s *t*-test was used for the statistical analysis of the immunoblotting densitometry of the TNF-treated groups compared with controls. Values of *p* < 0.05 were considered to be statistically significant and are presented as ∗*p* < 0.05, ∗∗*p* < 0.01, and ∗∗∗*p* < 0.001.

## Results

### Hematoxylin-Eosin Staining

**FIGURE 2:**
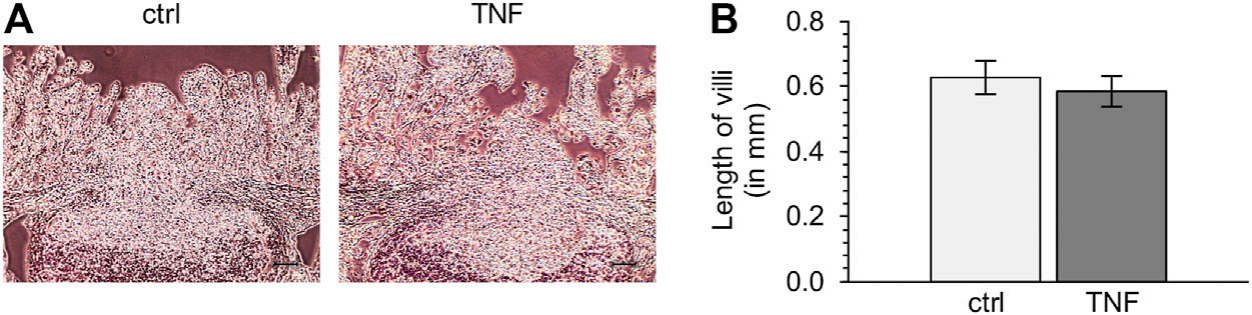
**(A)** Representative images of hematoxylin-eosin-stained tissues **(B)** Analysis of villus length of Peyer’s patch (PP)-surrounding villus epithelium (VE) after 10 h in the Ussing chamber with or without TNF (scale bar: 0.1 mm, *n* = 6).

To check the quality of tissue specimens after incubation for 10 h in Ussing chambers, we performed hematoxylin-eosin staining subsequent to the experiments. Both TNF-treated and untreated tissue samples showed no significant reduction of villus length compared with specimens fixed without incubation, and incubation with the cytokine did also not lead to significant changes in villus length compared with the control groups (ctrl: 0.63 ± 0.05 mm; TNF: 0.58 ± 0.05 mm; *p* = 0.57; unpaired *t*-test; Figure 2).

### Impact of TNF on Intestinal Epithelial Barrier Function of Porcine Peyer’s Patches and Villus Epithelium (Resistance and Permeability)

**FIGURE 3:**
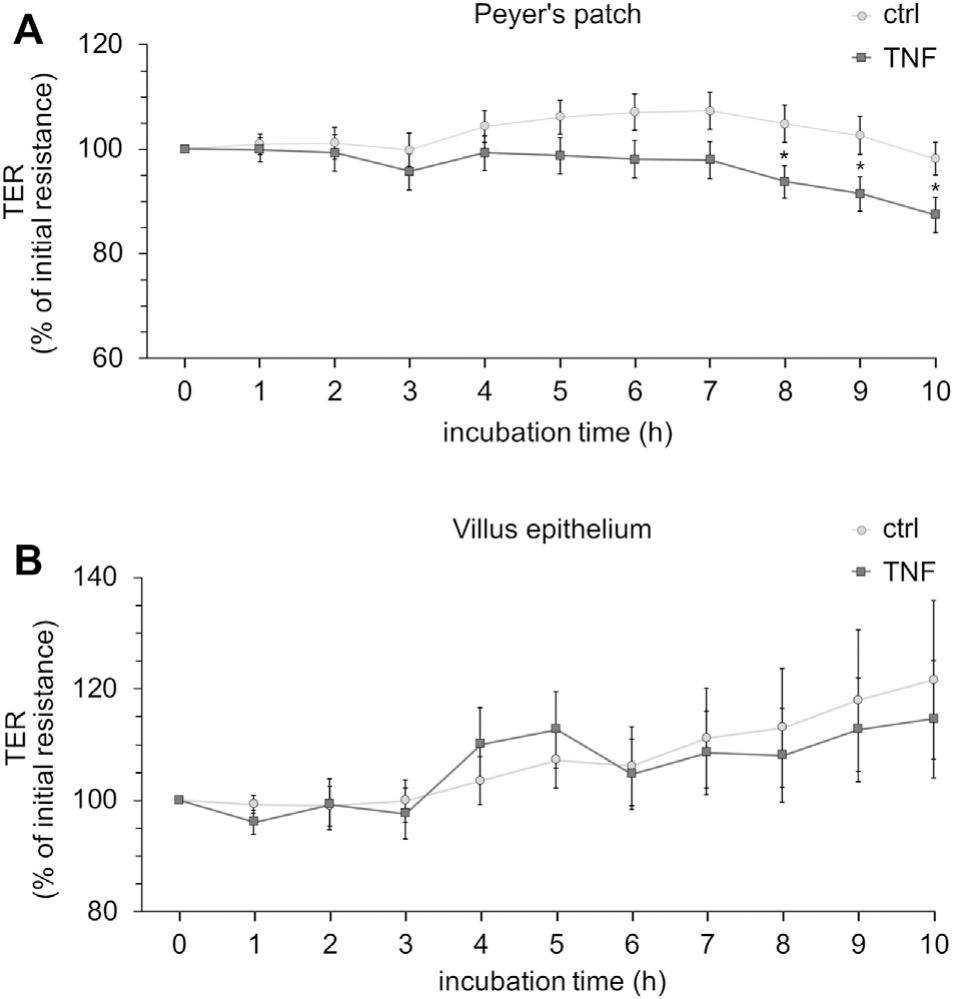
Time-dependent effect of TNF (5000 U/ml) on PP **(A)** or VE **(B)**. The cytokine was added to the serosal side of the tissues, and TER-values were recorded for up to 10 h. Data are shown as the percentage of initial resistance ± SEM (*n* = 8–23, *p < 0.05).

**FIGURE 4:**
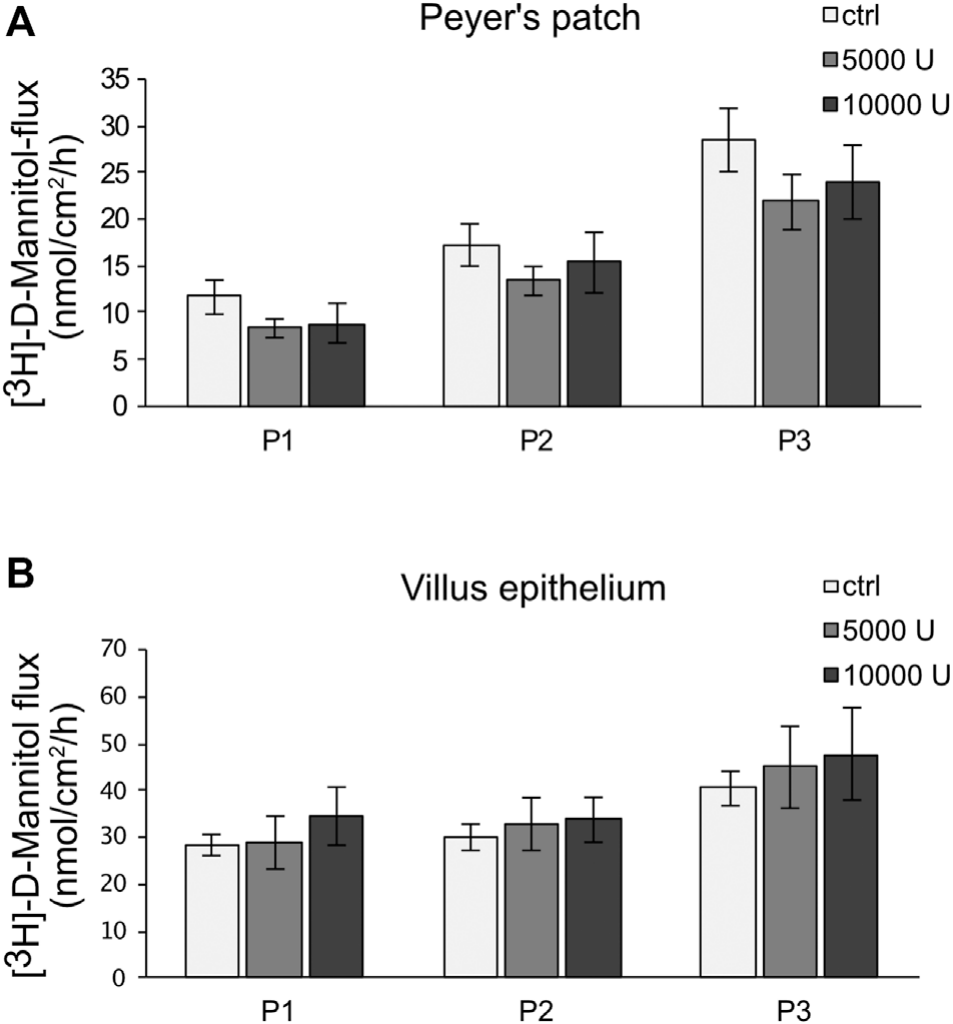
Paracellular permeability of PP **(A)** and VE **(B)** by using [^3^H]-D-Mannitol flux measurements. After an incubation of 8 h with TNF (5,000, 10,000 U/mL), flux measurements were carried out for 2 h, resulting in three flux periods of 40 min each. Data are presented as mean ± SEM (*n* = 7).

Ussing chamber experiments were carried out to examine the effect of the cytokine on immunologically active PP tissues compared with that on the surrounding VE. In PP tissue specimens, 1000 U/ml TNF caused no significant changes, whereas 5000 U/ml and 10,000 U/ml led to a decrease of TER after 8 h in the Ussing chamber compared with the control groups. All the following experiments were therefore performed with 5000 U/ml TNF. For clarity of presentation, only 5000 U/ml is presented in Figure 3A and is given as “TNF”. TER values at all concentrations are shown in Supplementary Table S1. The TER values of TNF-treated VE remained unaltered throughout the incubation (Figure 3B). The increasing trend of TER for both, controls and TNF-treated samples, could be a result of VE tissue being less robust during the time course of the experiment, although still vital. To investigate whether incubation with TNF led to altered paracellular permeability, unidirectional paracellular tracer flux measurements were carried out using 182 Da [^3^H]-D-Mannitol. Three flux periods of 40 min duration (P1—P3) were monitored. In specimens from PP tissues, neither 5000 U/ml nor 10,000 U/ml TNF led to significant changes in the apparent permeability during all three flux periods (Figure 4A). Furthermore, no significant changes in paracellular permeability were observed in neighboring VE tissue (Figure 4B). The flux rates of [^3^H]-D-Mannitol and the respective statistical analysis are shown in Supplementary Table S2.

### Changes in the Expression of TJ Proteins in PP and VE Attributable to TNF Treatment

**FIGURE 5:**
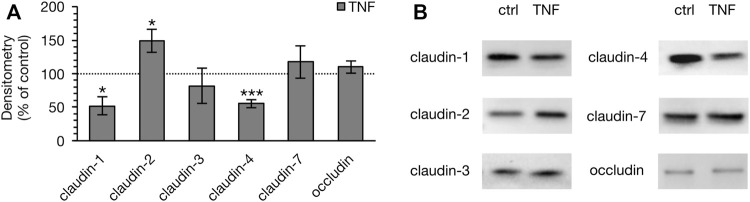
Densitometry **(A)** and representative Western blots **(B)** of TJ proteins in PP tissue after 10 h of incubation with 5000 U/ml TNF. A marked lower expression of claudin-1 and -4 was observed, whereas the expression of claudin-2 was stronger (*n* = 4–6, *p < 0.05, ***p < 0.001). The dotted line indicates the controls, which were set to 100% and compared to the TNF treated groups, respectively.

To determine whether the observed changes in the TER of PP tissues were attributable to the altered expression of TJ proteins, immunoblotting was performed subsequent to the Ussing chamber experiments (Figure 5). Densitometric analysis of Western blot bands revealed a remarkable decrease of claudin-1 after 10 h (52.40 ± 13.28%, *p* = 0.012, *n* = 4; unpaired *t*-test). In addition, we detected a major reduction of claudin-4 signal (55.84 ± 5.81%, *p* = 0.0003, *n* = 4). On the other hand, the pore-forming TJ protein claudin-2 had increased after 10 h (149.25 ± 16.77%, *p* < 0.026, *n* = 4). Treatment with TNF did not affect the expression of claudin-3 (82.40 ± 26.49%, *p* = 0.29, *n* = 5), claudin-7 (118.10 ± 24.12%, *p* = 0.47, *n* = 5), or occludin (110.47 ± 8.91%, *p* = 0.28, *n* = 4). In VE tissue, none of the above-mentioned TJ proteins showed any changes after treatment with the cytokine (data not shown).

### Confocal Laser Scanning Immunofluorescence Microscopy

**FIGURE 6:**
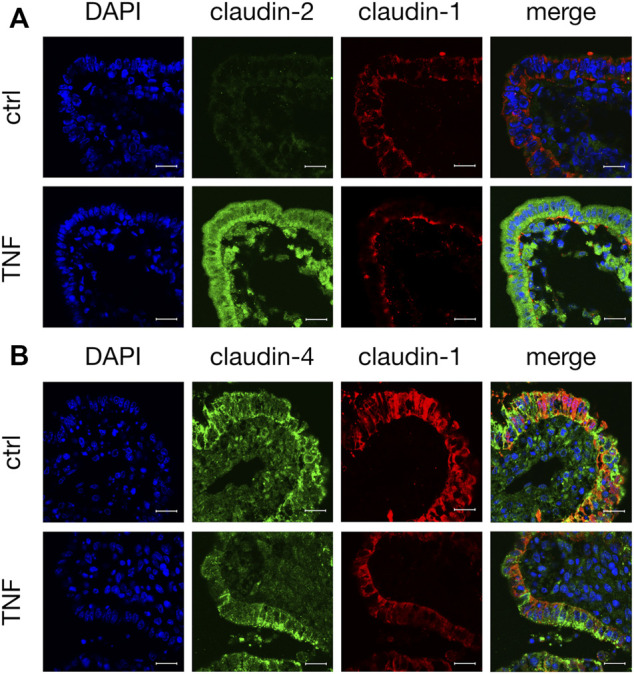
Immunohistological staining of affected TJ proteins after TNF incubation **(A)** Confocal laser scanning immunofluorescence microscopy of claudin-2 (green) and claudin-1 (red) after 10 h incubation of tissue in the Ussing chamber with or without 5000 U/ml TNF **(B)** Staining of PP FAE for claudin-4 (green) and claudin-1 (red). Cell nuclei were stained blue by DAPI (scale bar: 20 μm, *n* = 3, representative images).

Tissue samples were stained with antibodies raised against specific TJ proteins that had shown alterations in their expression when analyzed by Western blot. In the control tissue specimens after 10 h in the Ussing chamber, we observed a weak but specific claudin-2 signal (green, Figure 6A). Incubation with TNF strongly enhanced the expression of claudin-2, as the whole FAE of PP showed an intense signal for this TJ protein. The staining cells inside the FAE might be subjunctionally located signals of claudin-2, which were caused during protein biosynthesis. The same epithelial preparation revealed decreased signals and a disruption of claudin-1 after incubation with the cytokine, as the signal for claudin-1 was solely located in the basal membrane after TNF treatment. In untreated PPs, a strong claudin-1 signal was located in the apicolateral membrane (red, Figure 6A). Incubation with TNF also disturbed the strong paracellular signal of claudin-4. In contrast to claudin-1, the restricted claudin-4 signal was enriched in fragments at the apical region of the cells. The different orientations of the protein fragments of claudin-1 and -4 within the cells can be seen most clearly in the merged picture of the TNF-treated tissue in Figure 6B in which their colocalization is diminished.

### Expression Level of Specific TNF Receptors TNFR-1 and -2

**FIGURE 7:**
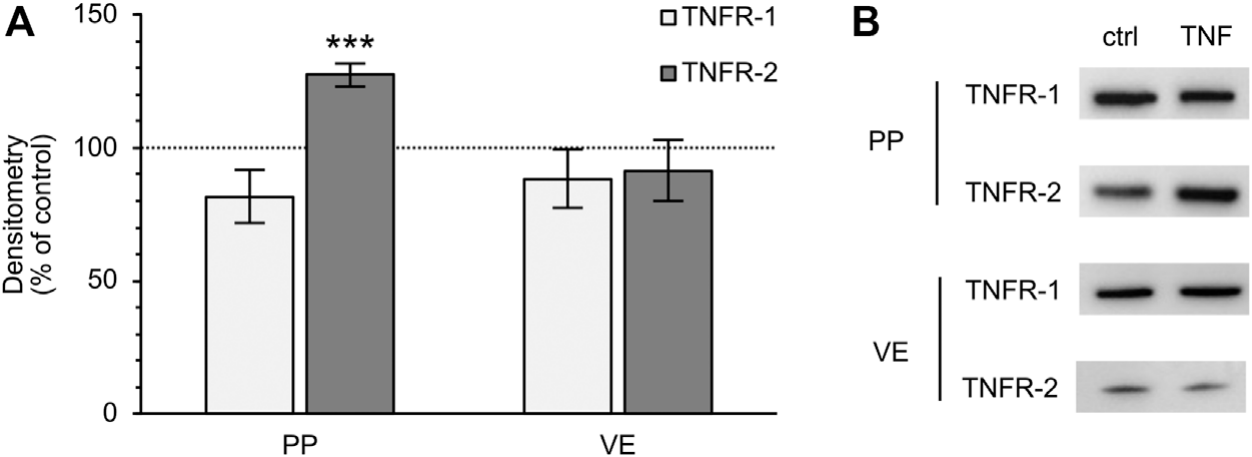
Densitometry **(A)** and representative Western blot bands **(B)** of TNFR-1 and -2 after 10 h incubation of PP FAE and VE tissue in the Ussing chamber with or without TNF. The expression of TNFR-2 increased in PP FAE tissue specimens after incubation with the cytokine, whereas the expression of TNFR-1 was not significantly altered (*n* = 4–7, ***p < 0.001). In VE samples, neither of the two receptors showed significant changes after exposure to TNF (*n* = 6–7). The controls were set to 100% and are depicted by the dotted line.

**FIGURE 8:**
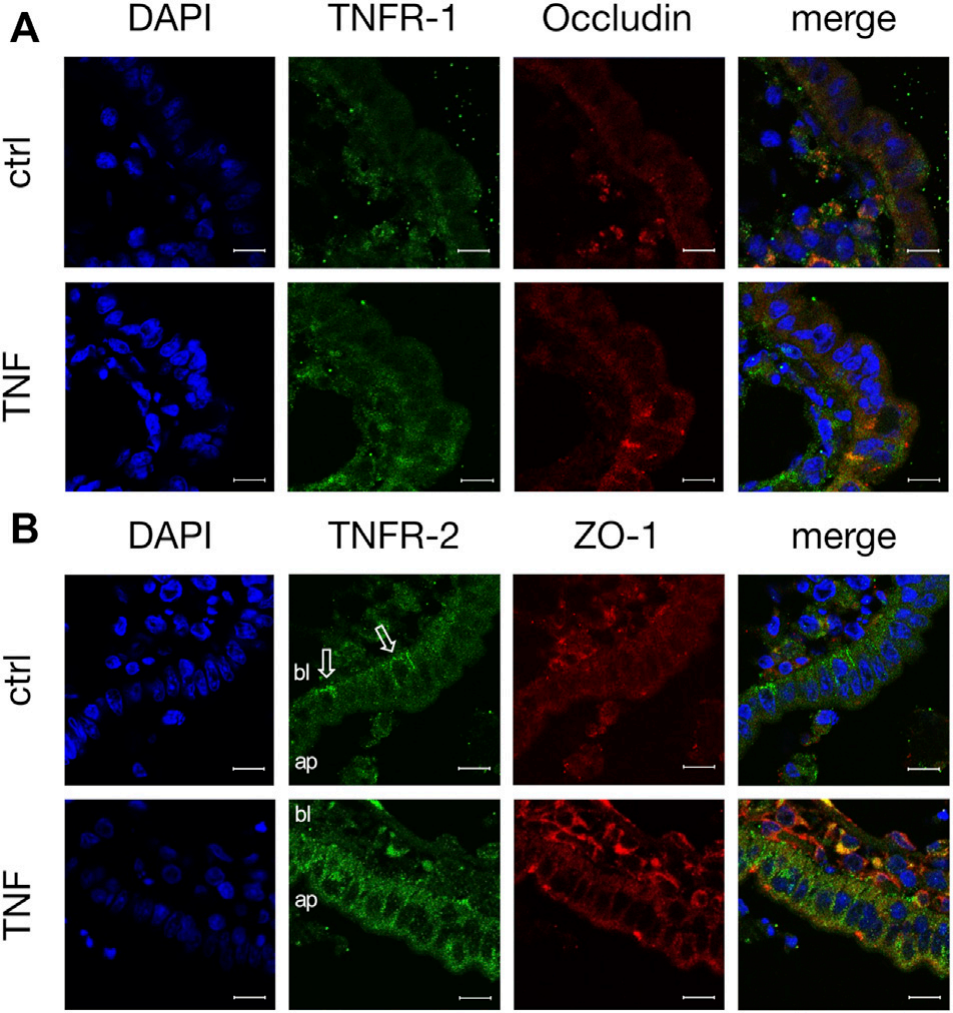
Immunohistological staining of specific TNF receptors **(A)** TNFR-1 (green) and occludin (red) after 10 h incubation of tissue with or without TNF **(B)** TNFR-2 (green) and ZO-1 (red) were stained for confocal laser scanning immunofluorescence microscopy. For better orientation, the basolateral side of FAE is labelled “bl”, and the apical part “ap”. White arrows indicate the basolateral signal for TNFR-2 in control tissues. DAPI was used for the staining of cell nuclei (scale bar: 40 μm, *n* = 3, representative images).

Because TNF can bind two specific receptors, namely TNFR-1 and -2, we examined which of these receptors mediates the TNF-induced changes in PP tissues. Western blot analysis was carried out with specific antibodies raised against TNFR-1 and -2. For densitometry, the Western blot bands were normalized to the total amount of protein, and controls were set to 100%. In FAE of PP, an increase of TNFR-2 was detected (TNF: 127.39 ± 4.48%; *p* < 0.0001, *n* = 7; unpaired *t*-test), whereas the expression of TNFR-1 was not significantly altered (TNF: 81.77 ± 10.13%; *p* = 0.12, *n* = 4, Figure 7). In VE, neither TNFR-1 (TNF: 88.38 ± 10.84%; *p* = 0.3, *n* = 7), nor TNFR-2 (TNF: 91.45 ± 11.44%; *p* = 0.47, *n* = 6), showed a significantly altered expression after exposure to TNF for 10 h. Confocal laser scanning immunofluorescence microscopy of PP tissue samples did not show any specific signals for TNFR-1, regardless of incubation with TNF (green, Figure 8A). In accordance with the results from the Western blot analysis, incubation with TNF did not alter the localization or signal intensity of occludin (red, Figure 8A). Only single cells of the FAE of untreated PP tissue specimens showed a specific signal for TNFR-2, which was only located in the basolateral membrane (green, white arrows, Figure 8B). After incubation with 5000 U/ml TNF for 10 h, almost all of the cells showed a strong signal for TNFR-2, which was located not only to the basolateral part, as seen for the control tissues, but also to the apical membrane. The signal for ZO-1 was not altered by incubation with the cytokine (red, Figure 8B).

### Signaling Experiments With ML-7

PP tissues were incubated with either TNF (5000 U/ml) or TNF + ML-7 (5000 U/ml + 7.5 μM), whereas controls were incubated with neither. The point at which the substances were added, TER values were set to 100% and recorded for 10 h. The TER of the TNF-treated group decreased to 72.12 ± 7.46%. Control groups exhibited TER values of 85.32 ± 6.68%, and the group treated with TNF + ML-7 showed resistance values of 83.32 ± 9.28% after 10 h incubation. All values were compared with initial resistance. Although a trend could be observed (Supplementary Figure S1), it did not reach significance (*p* = 0.46, *n* = 8; one-way ANOVA).

### Theophylline Challenge

By using theophylline, chloride secretion is stimulated in epithelial cells *via* cAMP, and therefore leads to an increased short-circuit current (I_sc_) indicating vitality of the tissue (Markov et al., 2014). An incubation with TNF did not influence the vitality of VE (ctrl: 56.25 ± 5.46 μA/cm^2^; 1000 U/ml: 58.63 ± 7.68 μA/cm^2^; 5000 U/ml: 55.38 ± 5.75 μA/cm^2^; 10,000 U/ml: 59 ± 8.07 μA/cm^2^, *p* = 0.98, *n* = 8; one-way ANOVA) and PP tissues (ctrl: 1.63 ± 0.75 μA/cm^2^; 1000 U/ml: 2 ± 1 μA/cm^2^; 5000 U/ml: 1.13 ± 0.77 μA/cm^2^; 10,000 U/ml: 2.25 ± 1.03 μA/cm^2^, *p* = 0.79, *n* = 8; Kruskal–Wallis test, Supplementary Figure S2), though. Regarding the outcome of the vitality tests carried out, a cut-off TER for vital tissue was determined for the following experiments, indicated by respective VE values representative for both tissues of one preparation step.

### Incubation of Porcine Intestinal Epithelial Cell Line With TNF

**FIGURE 9:**
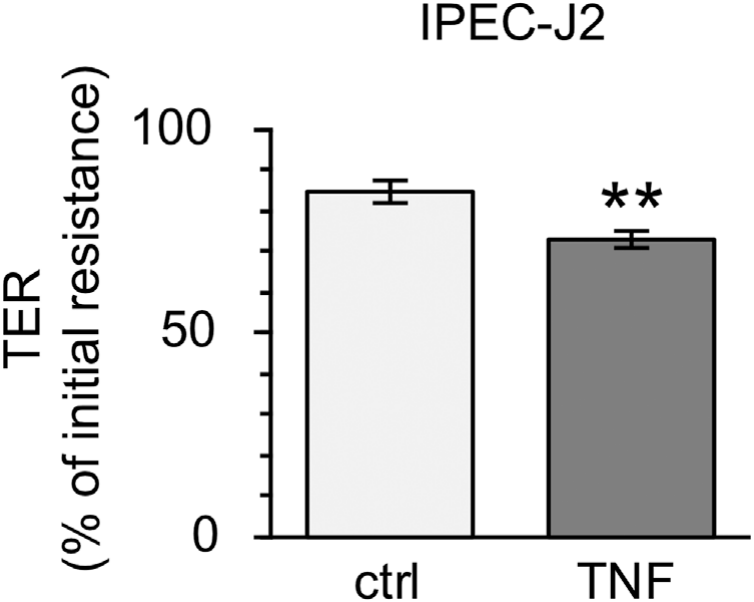
Incubation of IPEC-J2 cells with 5000 U/ml TNF for 10 h. The cytokine was added to the basolateral compartment of the cell inserts. A marked decrease in TER, comparable with results seen in PP tissue specimens, was observed (*n* = 23, **p < 0.01).

Because the incubation of PP tissues with TNF led to notable changes in epithelial barrier function, further analyses with the non-transformed cell line IPEC-J2 were performed. After incubation with 5000 U/ml for 10 h, the TER of the confluent cell monolayers was reduced comparable to the changes seen in Ussing chamber trials (ctrl: 84.63 ± 2.79%; TNF: 73.09 ± 2.21%, *p* = 0.002, *n* = 23; unpaired *t*-test; Figure 9).

## Discussion

Previous studies have focused on the effects of TNF on the intestinal epithelial barrier, and, in particular, on the disturbance of TJ proteins (Barmeyer et al., 2017). Nevertheless, the impact that TNF might have, especially on the barrier properties of the FAE of porcine PP, remains unknown.

The porcine PP contributes to the GALT and represents one of the first lines of intestinal immune defense (Radloff et al., 2017). By presenting luminal antigens to the immune cells, the FAE, which covers PPs, strictly narrows and regulates the contact of the immune system with exogenous substances and plays a key role in the regulation of immune processes (Gebert et al., 1996). An exposure of PPs to cytotoxic substances resulted in an increased expression of the proinflammatory cytokine TNF (Andreev-Andrievskiy et al., 2021; Takasu et al., 2021). Therefore, the barrier function of PPs and its regulation is profoundly biomedically significant, as the GALT controls immune processes, such as food allergies, cancer development or intestinal inflammation (Ishii et al., 2010; Nakajima-Adachi et al., 2014; Fujimoto et al., 2015).

Claudin-1 has been described as a major sealing TJ protein in a variety of tissues including intestine, kidney, pleura, skin, and the blood-brain barrier (Amasheh et al., 2011; Rosenthal et al., 2012; Markov and Amasheh, 2014). In several publications, enhanced TNF levels have been shown to decrease the expression of claudin-1, explaining the disturbed barrier function after exposure to the cytokine (Bruewer et al., 2003; Epple et al., 2009). However, differential effects have been described: incubation with TNF can also lead to the increased expression of claudin-1 (Amasheh et al., 2010; Shiozaki et al., 2012; Bhat et al., 2016). One explanation for this apparently paradoxical effect of claudin-1 might be that, even although an increased expression of claudin-1 occurs, its functional contribution remains questionable if it is not localized in apicolateral TJs (Poritz et al., 2011). The effect might be the result of a counter-regulatory induction effect, with claudin-1 not being properly trafficked into the functionally relevant TJ complexes. However, in our current study, a decrease of claudin-1 was observed after incubation with TNF, which is in accordance with the disrupted barrier function in porcine PP FAE. Therefore, this effect might explain barrier disruption in many inflammatory processes.

In contrast to the sealing function of claudin-1, claudin-2 is a pore-forming TJ protein allowing the paracellular passage of water and cations that are smaller than 182 Da (Amasheh et al., 2002; Rosenthal et al., 2010). In several gastrointestinal disorders, such as IBDs or gastrointestinal carcinomas, the expression of claudin-2 is increased (Aung et al., 2006; Mankertz and Schulzke, 2007). An increase of claudin-2 in the intestine results in a drop in TER and in increased paracellular permeability (Ahmad et al., 2014; Wang et al., 2017; Markov et al., 2019). This is in accordance with our findings in the current study, as we have detected an increase of claudin-2 after 10 h incubation with TNF.

Another TJ protein with a major sealing function is claudin-4. Radloff *et al.* have shown higher expression of claudin-4 in the porcine PPs compared with the surrounding VE, in agreement with the stronger barrier properties of the FAE (Radloff et al., 2017). Therefore, the decrease of claudin-4 in our study contributes to the disturbed sealing function in FAE after the TNF challenge, representing a weakened barrier in PPs during intestinal inflammation. The expression of claudin-3, -7, and occludin, which are also barrier-forming TJ proteins, was not significantly changed by TNF in our study.

In contrast to the strong decrease of TER, the flux of [^3^H]-D-Mannitol, representing the paracellular permeability, remained unchanged. Because of the differential regulation of permeability pathways, it is not unusual that the paracellular permeability is unaltered, whereas TER has changed (Schoultz and Keita, 2020). The underlying reason is the activation of barrier permeability by two different TJ pathways, namely the pore and leak pathway (Turner, 2009). The high-capacity pore pathway, which is size- and charge-selective, enables solutes smaller than 4 Å in radius to cross the paracellular route (Van Itallie et al., 2008). Molecules larger than 4 Å might pass the paracellular barrier *via* the low-capacity leak pathway. The knockdown of ZO-1 or occludin and disrupted cell-to-cell contacts have been reported to increase the leak pathway (Anderson and Van Itallie, 2009). Furthermore, with cytokines such as TNF, the paracellular flux through the leak pathway seems to be increased. In our experiments, the incubation of porcine PP with TNF led to a decrease of TER after 8 h, whereas the paracellular flux was not significantly changed. This might be attributable to the molecular weight of [^3^H]-D-Mannitol (182 Da), which is slightly too big to pass the claudin-2-formed pore (Amasheh et al., 2002). The results are also in accordance with the functional properties of claudin-4 sealing the paracellular pathway against the passage of ions (Van Itallie et al., 2001). Non-significant effects on mannitol permeability could also reflect an early or initial TNF effect, as in different epithelial cell models, permeability changes were seen after incubation with TNF for at least 24 h (Ma et al., 2004; Poritz et al., 2011).

An induced leak to Na^+^ and Cl^−^ would represent a higher permeability of the high-capacity pore pathway, and our model most likely represents an early pathomechanism of barrier perturbation by TNF, i) affecting the secretory mechanisms associated with diarrhea and ii) initiating to stronger barrier perturbation in synergism with other proinflammatory cytokines, e.g., Interferon γ (IFNγ; Wang et al., 2006). In accordance, TNF only affecting the ion permeability has been observed previously (Marano et al., 1998).

In previous studies of TNF effects, a pre- or co-incubation with IFNγ has been carried out (Wang et al., 2005; Amasheh M. et al., 2009; Cao et al., 2013). The reason for this step is the ability of IFNγ to increase the expression of specific TNF-receptors (TNFR), hence leading to a faster effect of TNF (Wang et al., 2006). Though, whether the observed changes are a pure effect of TNF or whether it is linked to the incubation with IFNγ remains unclear. Here, however, we have analyzed the single (and direct) effect of TNF, as in our recent publication, a long term-effect of TNF on the non-transformed porcine cell line IPEC-J2 has been shown without prior or simultaneous IFNγ incubation, representing the selective effect of TNF (Droessler et al., 2021).

The TNF effect can be induced by activation of the specific TNF receptors TNFR-1 or -2. Therefore, we carried out additional approaches in order to determine whether incubation with TNF leads to changes in the expression of TNF receptors in PP FAE in comparison with VE. Our study has revealed the main relevance of TNFR-2 in FAE, whereas in VE no significant effects on TNF receptors were observed.

Although TNFR-1 is constitutively found in most tissues, TNFR-2 plays a major role in cells of the immune system (Wajant et al., 2003). TNFR-2 has been described as being involved particularly in immune modulation and tissue regeneration, whereas TNFR-1 on the contrary is mostly associated with inflammation. Another major difference between the two receptors is the ability of TNFR-1 to become activated by soluble and membrane-bound TNF, while TNFR-2 can only be activated by membrane-bound TNF (Fischer et al., 2015). Selective targeting of TNFR-1 and -2 is currently of great interest for therapeutic approaches, as the employment of anti-TNF drugs might cause severe side-effects. Although the genetic deletion of TNFR-1 in animal models leads to an absence of TNF-induced diseases, the deletion of TNFR-2 worsens the disorders (Fischer et al., 2020). The findings that the effects of TNF in porcine PP are mediated by TNFR-2 emphasizes the importance of FAE as being the first step of the intestinal immune response. The predominant TNF-signaling *via* TNFR-2 might also be the reason for the lack of an effect of the inhibitor ML-7 on porcine PP, as previous studies have shown that the blocking of MLCK prevents the strong increase of TNFR-1 after TNF-incubation (Droessler et al., 2021).

To strengthen our findings further, additional experiments were carried out using the non-transformed cell-line IPEC-J2. This porcine jejunal cell-line has recently been used for analysis of TNF effects on the intestinal barrier function. Significant effects on transepithelial resistance and paracellular flux of [^3^H]-D-Mannitol having been seen after incubation of the cells with 1000 U/ml TNF for 48 h (Droessler et al., 2021). In the present study, incubation of these cells with 5000 U/ml for 10 h has revealed changes in transepithelial resistance, in agreement with our findings in the Ussing chamber experiments. Although IPEC-J2 cells are a suitable model for analysis of small intestine and these cells represent many aspects of the VE, the different outcome of IPEC-J2 cells and VE in our manuscript indicates that this cell model needs to be compared carefully in different experimental approaches. Therefore, experiments with IPEC-J2 in our current study were performed under the same conditions regarding concentration and time course, to allow a better comparison of the results with previous approaches.

Our aim has been to analyze PP and neighboring VE *ex vivo,* as the tissue samples are more comparable with the intestine *in vivo.* Moreover, the PP samples allow us to investigate the direct effect of TNF on FAE. By employing the Ussing chamber technique, we performed a detailed analysis of the effects of TNF on TER for up to 10 h. Subsequently, tissues were stained with hematoxylin-eosin, and the size of PPs and the length of the surrounding villi were measured and compared between the control and TNF-treated groups. Apoptosis could be excluded as a reason for the observed changes, as the tissue samples still responded to vitality tests carried out using theophylline. Because no significant alterations in tissue quality after incubation with TNF were observed, and no evidence for apoptosis could be detected, other mechanisms for the drop in TER needed to be explored, with special attention being given to barrier perturbation, as described in detail above. It cannot be ruled out, that a longer incubation with TNF might also affect the functional barrier properties of VE additional to changes in PP FAE, as intestinal inflammation like in PWD go also in accordance with a disturbed morphology of VE in porcine (Chen et al., 2020).

In our current study, we have been able to confirm the functional and molecular differences of PP and VE under intestinal inflammatory conditions. The importance of the porcine FAE as a first step in intestinal immunological defense has been established, as only PP tissue specimens reacted to the addition of the proinflammatory cytokine TNF with changes in barrier function, TJ protein composition, and the expression of TNFR-2, whereas VE did not respond. The outcome of our current study might allow further approaches focusing on the identification and analysis of counter-regulatory, beneficial, and therefore possibly therapeutic and preventative compounds for administration under inflammatory intestinal conditions.

## Data Availability

The raw data supporting the conclusion of this article will be made available by the authors on request.

## Abbreviations

FAE, follicle-associated epithelium; GALT, gut-associated lymphatic tissue; IFNγ, Interferon gamma; I_SC,_ short-circuit current; PFA, paraformaldehyde; PP, Peyer’s patch; TER, transepithelial resistance; TJ, tight junction; TNF, tumor necrosis factor; TNFR, tumor necrosis factor receptor; VE, villus epithelium.
