# Supplementary material for: Barrier Perturbation in Porcine Peyer’s Patches by Tumor Necrosis Factor is Associated With a Dysregulation of Claudins
Source: Front Physiol. 2022 May 30;13:889552. doi: 10.3389/fphys.2022.889552 (PMC9189282; doi:10.3389/fphys.2022.889552)
Supplement: Supplementary file 1 [file Table1.DOCX]

**Table 1.** Changes in TER after treatment of PP or VE with different concentrations of TNF (1000, 5000 and 10000 U/mL)^1^

|  | TNF  (U/mL) | | % of initial resistance | | | | | | | | | | | | | | | | | | |
| --- | --- | --- | --- | --- | --- | --- | --- | --- | --- | --- | --- | --- | --- | --- | --- | --- | --- | --- | --- | --- | --- |
|  |  |  | 1 h | 2 h | | 3 h | | 4 h | | 5 h | | 6 h | | 7 h | | 8 h | | 9 h | | 10 h | |
| PP | **0** | 101 ± 2 n = 28 | | 101 ± 3 n = 28 | 100 ± 3 n = 28 | | 104 ± 3 n = 28 | | 106 ± 3 n = 28 | | 107 ± 4 n = 23 | | 107 ± 4 n = 23 | | 105 ± 4 n = 23 | | 103 ± 4 n = 23 | | 98 ± 3 n = 23 | |  |
|  | **1000** | 99 ± 2 n = 8 | | 98 ± 3 n = 8 | 95 ± 3 n = 8 | | 99 ± 3 n = 8 | | 104 ± 3 n = 8 | | 103 ± 4 n = 8 | | 104 ± 4 n = 8 | | 100 ± 4 n = 8 | | 94 ± 4 n = 8 | | 91 ± 3 n = 8 | |  |
|  | **5000** | 100 ± 2 n = 28 | | 99 ± 4 n = 28 | 96 ± 3 n = 28 | | 99 ± 3 n = 28 | | 99 ± 3 n = 28 | | 98 ± 4 n = 23 | | 98 ± 4 n = 23 | | 94 ± 3* n = 23 | | 91 ± 3* n = 23 | | 87 ± 3* n = 23 | |  |
|  | **10000** | 96 ± 1 n = 25 | | 94 ± 2 n = 25 | 91 ± 2 n = 25 | | 95 ± 1 n = 25 | | 96 ± 2 n = 25 | | 96 ± 2 n = 20 | | 96 ± 2* n = 20 | | 93 ± 2* n = 20 | | 90 ± 3* n = 20 | | 86 ± 3* n = 20 | |  |
| VE | **0** | 99 ± 2 n = 24 | | 99 ± 4 n = 24 | 100 ± 4 n = 24 | | 103 ± 4 n = 24 | | 107 ± 5 n = 24 | | 106 ± 7 n = 19 | | 111 ± 9 n = 19 | | 113 ± 11 n = 19 | | 118 ± 13 n = 19 | | 122 ± 14 n = 19 | |  |
|  | **1000** | 97 ± 3 n = 8 | | 96 ± 5 n = 8 | 96 ± 6 n = 8 | | 100 ± 6 n = 8 | | 104 ± 8 n = 8 | | 101 ± 8 n = 8 | | 98 ± 8 n = 8 | | 97 ± 9 n = 8 | | 98 ± 10 n = 8 | | 99 ± 10 n = 8 | |  |
|  | **5000** | 96 ± 2 n = 24 | | 99 ± 5 n = 24 | 98 ± 5 n = 24 | | 110 ± 7 n = 24 | | 113 ± 7 n = 24 | | 105 ± 6 n = 19 | | 109 ± 7 n = 19 | | 108 ± 8 n = 19 | | 113 ± 9 n = 19 | | 115 ± 11 n = 19 | |  |
|  | **10000** | 98 ± 2 n =24 | | 100 ± 4 n = 24 | 100 ± 4 n = 24 | | 109 ± 5 n = 24 | | 108 ± 6 n = 24 | | 102 ± 8 n = 19 | | 105 ± 10 n = 19 | | 103 ± 10 n = 19 | | 105 ± 12 n = 19 | | 106 ± 13 n = 19 | |  |

## ^1^ Values are presented in mean ± SEM, * p < 0.05. Before addition of the cytokine, TER values were normalized to 100 % and data were compared with the control group for each time point, respectively.
