# Supplementary figures and images for: Barrier Perturbation in Porcine Peyer’s Patches by Tumor Necrosis Factor is Associated With a Dysregulation of Claudins
Source: Front Physiol. 2022 May 30;13:889552. doi: 10.3389/fphys.2022.889552 (PMC9189282; doi:10.3389/fphys.2022.889552)

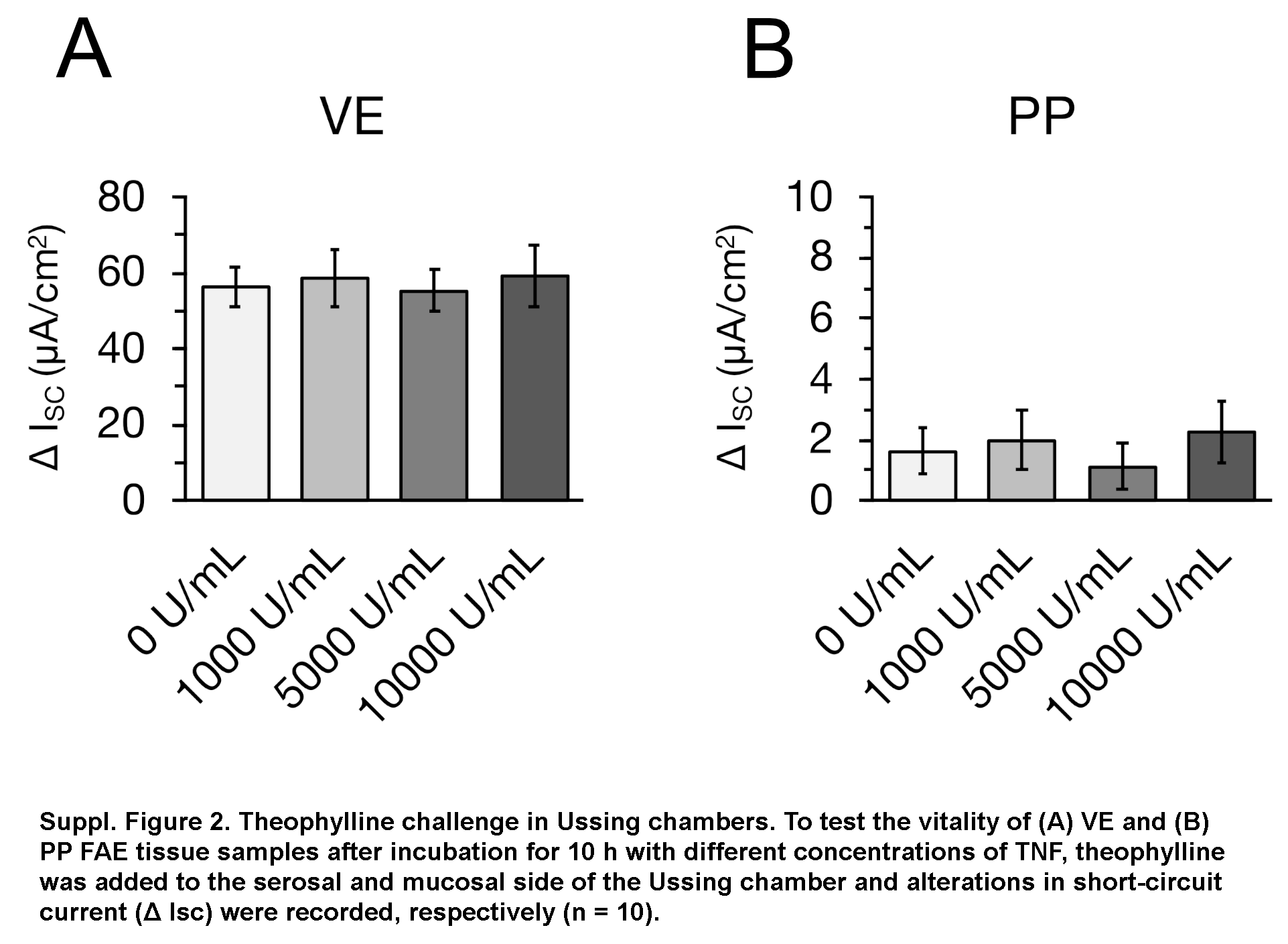

Supplement: Supplementary file 2 [file Image2.tif]

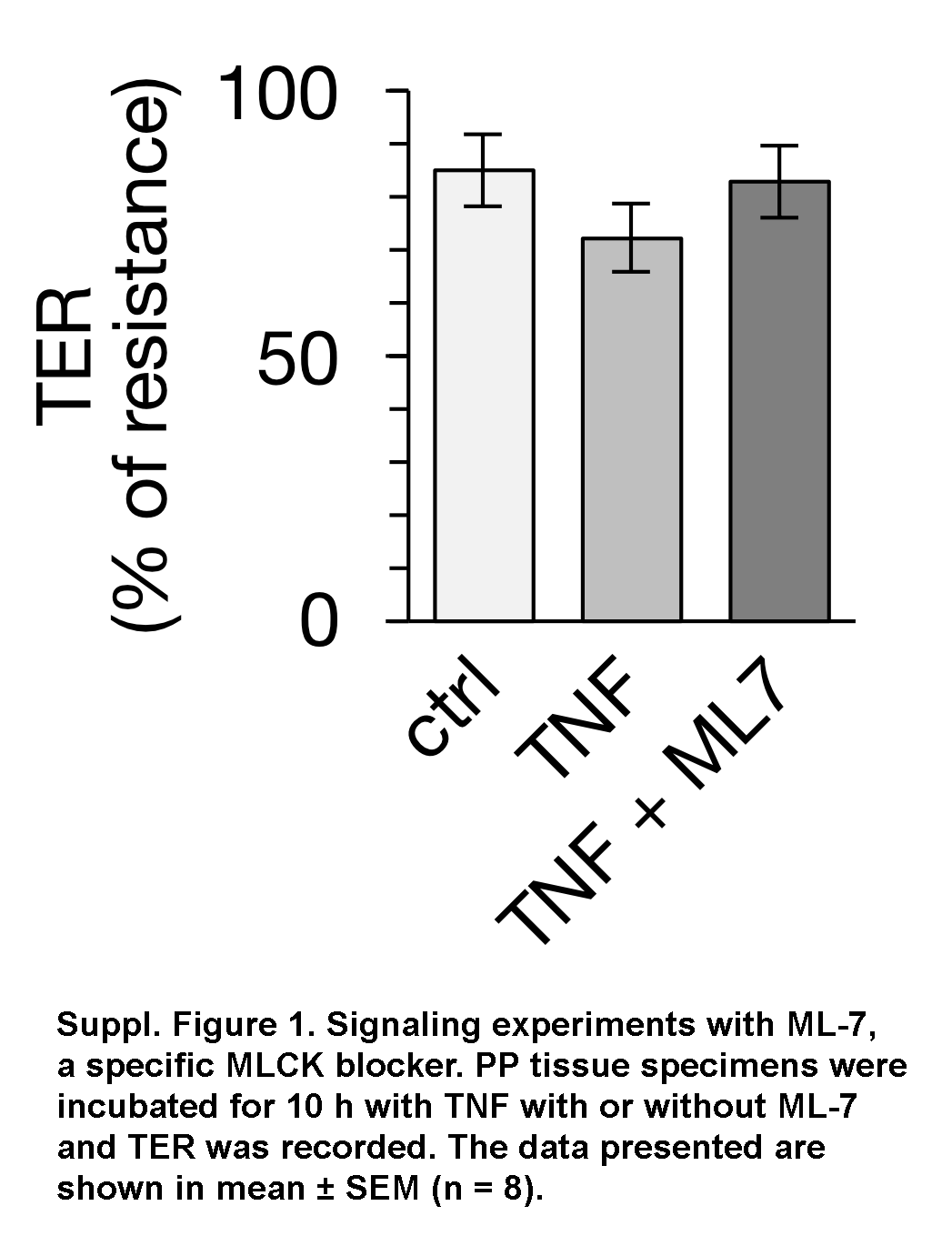

Supplement: Supplementary file 3 [file Image1.tif]
