# Supplementary material for: Barrier Perturbation in Porcine Peyer’s Patches by Tumor Necrosis Factor is Associated With a Dysregulation of Claudins
Source: Front Physiol. 2022 May 30;13:889552. doi: 10.3389/fphys.2022.889552 (PMC9189282; doi:10.3389/fphys.2022.889552)
Supplement: Supplementary file 4 [file Table2.docx]

**Table 2.** [^3^H]-D-Mannitol flux rates of three flux periods of 40 minutes in PP or VE during incubation with TNF (5000 or 10000 U/mL)^1^

|  | TNF  (U/mL) | [^3^H]-D-Mannitol flux rates  (nmol/cm^2^/h) | | |
| --- | --- | --- | --- | --- |
|  |  | P1 | P2 | P3 |
| PP | **0** | 11.7 ± 1.8 n = 7 | 17.3 ± 2.2 n = 7 | 28.4 ± 3.4 n = 7 |
|  | **5000** | 8.4 ± 1 n = 7 | 13.4 ± 1.4 n = 7 | 21.9 ± 3.0 n = 7 |
|  | **10000** | 8.8 ± 2.1 n = 7 | 15.4 ± 3.2 n = 7 | 23.8 ± 4 n = 7 |
|  | one-way ANOVA | F (2,18) = 1.13, *p* = 0.35 | F (2,18) = 0.68, *p* = 0.52 | F (2,18) = 0.93, *p* = 0.41 |
| VE | **0** | 28.4 ± 2.3 n = 7 | 29.9 ± 2.7 n = 7 | 40.5 ± 3.8 n = 7 |
|  | **5000** | 28.7 ± 5.7 n = 7 | 32.9 ± 5.6 n = 7 | 45 ± 8.9 n = 7 |
|  | **10000** | 34.6 ± 6.3 n = 7 | 33.9 ± 4.8 n = 7 | 47.7 ± 9.8 n = 7 |
|  | Kruskal-Wallis test | H (2) = 1.07, *p* = 0.59 | H (2) = 0.74, *p* = 0.69 | H (2) = 0.14 *p* = 0.93 |

*^1^ Values are presented in mean ± SEM.*
